# On the hunt for the alternate host of *Hemileia vastatrix*

**DOI:** 10.1002/ece3.5755

**Published:** 2019-11-23

**Authors:** Athina Koutouleas, Hans Jørgen Lyngs Jørgensen, Birgit Jensen, Jens‐Peter Barnekow Lillesø, Alexander Junge, Anders Ræbild

**Affiliations:** ^1^ Department of Geosciences and Natural Resource Management University of Copenhagen Frederiksberg C Denmark; ^2^ Department of Plant and Environmental Sciences and Copenhagen Plant Science Centre University of Copenhagen Frederiksberg C Denmark; ^3^ Faculty of Health and Medical Sciences Novo Nordisk Foundation Center for Protein Research University of Copenhagen Copenhagen N Denmark

**Keywords:** coffee leaf rust, disease cycle, *Hemileia vastatrix*, hypothetical alternate host ranking

## Abstract

Coffee leaf rust (CLR), caused by the fungal pathogen *Hemileia vastatrix*, has plagued coffee production worldwide for over 150 years. *Hemileia vastatrix* produces urediniospores, teliospores, and the sexual basidiospores. Infection of coffee by basidiospores of *H. vastatrix* has never been reported and thus far, no alternate host, capable of supporting an aecial stage in the disease cycle, has been found. Due to this, some argue that an alternate host of *H. vastatrix* does not exist. Yet, to date, the plant pathology community has been puzzled by the ability of *H. vastatrix* to overcome resistance in coffee cultivars despite the apparent lack of sexual reproduction and an aecidial stage. The purpose of this study was to introduce a new method to search for the alternate host(s) of *H. vastatrix*. To do this, we present the novel hypothetical alternate host ranking (HAHR) method and an automated text mining (ATM) procedure, utilizing comprehensive biogeographical botanical data from the designated sites of interests (Ethiopia, Kenya and Sri Lanka) and plant pathology insights. With the HAHR/ATM methods, we produced prioritized lists of potential alternate hosts plant of coffee leaf rust. This is a first attempt to seek out an alternate plant host of a pathogenic fungus in this manner. The HAHR method showed the highest‐ranking probable alternate host as *Psychotria mahonii*, *Rubus apetalus*, and *Rhamnus prinoides*. The cross‐referenced results by the two methods suggest that plant genera of interest are Croton, Euphorbia, and Rubus. The HAHR and ATM methods may also be applied to other plant–rust interactions that include an unknown alternate host or any other biological system, which rely on data mining of published data.

## Graphical abstract

This hypothesis piece probes a 150‐year‐old question… “is there an alternate host of the fungal pathogen *Hemileia vastatrix*?” We use comprehensive geographical botanical data and automated text mining to narrow down the possibilities. Credit: Carvalho et al. [CC BY 2.5 (https://creativecommons.org/licenses/by/2.5)]

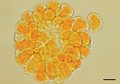

## 1 INTRODUCTION

The genus *Coffea* is composed of over one hundred species, which grow wild in equatorial Africa and Madagascar (Lashermes, Bertrand, & Ettienne, 2009; McCook, 2006). One of the major diseases threatening coffee production is coffee leaf rust (CLR), caused by the biotrophic rust fungus, *Hemileia vastatrix* Berk. & Broome (Basidiomycota, Pucciniales) (Berkeley & Broome, 1869; Toniutti et al., 2017). Wild coffee species and *H. vastatrix* have co‐evolved for hundreds of years in equatorial Africa, and the fungus was restricted to this continent up until the mid‐nineteenth century (McCook, 2006). Today, *H. vastatrix* is able to infect all known cultivated species in the genus *Coffea*, albeit at different levels of severity (McCook, 2006). Since the first significant outbreak in Sri Lanka (Ceylon) in 1869, almost the entire world's coffee producing zones have reported coffee leaf rust attacks, resulting in up to 40% annual yield losses (Arneson, 2000; Kumar, Sreedharan, Shetty, & Parvatam, 2016; McCook, 2006).

**Figure 1:**
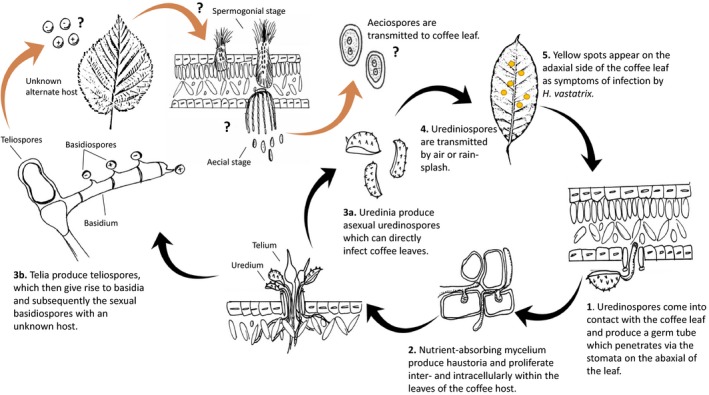
Disease cycle of *Hemileia vastatrix*. The black arrows indicate currently known uredinial stage (modified from Arneson, 2000), and orange arrows indicate the hypothesized aecial stage occurring on an unknown host plant(s)

*Hemileia vastatrix* penetrates coffee leaves via the stomatal openings and grows nutrient‐absorbing mycelium through the leaf mesophyll. Vibrant bouquet‐shaped, orange uredinia and telia are produced on the abaxial side of the coffee leaves (Arneson, 2000; Kumar et al., 2016). Uredinia give rise to urediniospores, which are dikaryotic and the only reported means of propagation for *H. vastatrix* (Arneson, 2000; Carvalho, Fernandes, Carvalho, Barreto, & Evans, 2011) (Figure 1). Dry urediniospores can survive up to 6 weeks on detached plant tissue, but will only germinate again in the presence of rain or heavy dew (Arneson, 2000). Under cool, dry conditions, the telia give rise to the two more elusive spore types: teliospores and subsequently basidiospores (Arneson, 2000; Coutinho, Rijkenberg, & Asch, 1995). Teliospores are two‐celled, thick‐walled and consist of dikaryotic cells (Schumann & Leonard, 2000). Teliospores produce basidia, which then develop four haploid basidiospores (Arneson, 2000; Coutinho et al., 1995) (Figure 1). In most rust fungi, only the teliospores are capable of long‐term survival away from a living host plant (Schumann & Leonard, 2000). By producing both asexual and sexual spore types, rust fungi increase the chance of transmission to multiple hosts (Shattock & Preece, 2000). For this reason, many rusts are observed to have complex disease cycles with different spore types or reproductive structures being defined as either macrocyclic (producing five spore types: spermatia, aeciospores, urediniospores, teliospores, and basidiospores) or microcyclic (species often lacking aeciospores and urediniospores, with or without spermatia) (Shattock & Preece, 2000). The sexual stage of a rust fungus' life cycle is of particular importance, because it facilitates the rise of new genotypes via recombination (Shattock & Preece, 2000). Despite the long history of CLR and the wide interest of the plant pathology community, critical aspects of the disease cycle of *H. vastatrix* remain unclear (Carvalho et al., 2011). Some have hypothesized that *H. vastatrix* is a heteroecious rust, thus requiring two hosts for the completion of the disease cycle (Gopalkrishnan, 1951; Petersen, 1974). The fact that basidiospores do not re‐infect coffee supports this theory (Gopalkrishnan, 1951). Yet, an alternate host of *H. vastatrix* has never been reported. It has been postulated that the basidiospores of *H. vastatrix* are remnants of an earlier rust ancestor and no longer utilized by the fungus (Arneson, 2000; Waller, 1982). However, others argue that the preservation of the basidiospores in the observed disease cycle provides evidence for a viable, alternate host of *H. vastatrix* (Petersen, 1974). Others have speculated that based on Tranzschel's Law (Shattock & Preece, 2000), the alternate host of *H. vastatrix* is an orchid (Rodrigues, 1990).

One of the earliest attempts to re‐infect coffee leaves with the “sporidia” (aka. basidiospores) arising from *H. vastatrix* teliospores was described as an “utter failure” (Ward, 1882). Since then, there have apparently been no reports of infection by *H. vastatrix* basidiospores in any plant species. This leads us to ask why this spore type is being produced by the fungus at all? There are examples of autoecious (single host) rust fungi, which can infect the same host with all spore types, such as the macrocyclic rust *Puccinia helianthi*, the causal agent of sunflower rust (Hiratsuka & Sato, 1982). However, it is most often observed that basidiospores do not infect the same plant species from which they originated (Kolmer, Ordonez, & Groth, 2009; Petersen, 1974). This implies that there is a high likelihood of an unrelated, alternate host, which *H. vastatrix* could infect to produce spermogonia and later aecia to complete the disease cycle (Figure 1).

Furthermore, a historical report by a British expedition to Sri Lanka in 1882 led to specimen collections of “jungle leaves” including palms, dicots, ferns, and grasses that exhibited the characters of *H. vastatrix* being chlorotic yellow, “pin‐spots” *(*Ferguson & Ferguson, 1882). However, upon later scientific examination, no signs of *H. vastatrix* could be confirmed (Ferguson & Ferguson, 1882). To the best of our knowledge, no subsequent studies to search for the possible aecial hosts of *H. vastatrix* have been published. Another possibility is that multiple host species of *H. vastatrix* exist, as with the *Cronartium* species *C. flaccidum* and *C. ribicola*. These rust pathogens have been reported to infect eight diverse host plants from six different families in greenhouse inoculation experiments (Kaitera, Hiltunen, & Hantula, 2017).

Modern coffee breeding and cultivation have led to a continuous evolution exertion on *H. vastatrix* by selection for resistance to CLR in commercial *Coffea* spp. cultivars (Silva, Várzea, Paulo, & Batista, 2018). Today, more than 50 races of *H. vastatrix* are known (Talhinhas et al., 2017). This is an inexplicable evolution for a pathogen that supposedly only utilizes clonal reproduction (Silva et al., 2018). Some reports have started to emerge, hypothesizing that the different races of *H. vastatrix* are the result of cryptosexuality, that is, the occurrence of hidden sexual reproduction within the urediniospores (Carvalho et al., 2011). However, these new findings would not explain the ability of *H. vastatrix* to produce basidiospores from the teliospores. Another hypothesis relating to the CLR outbreaks in Central America are based on primary host density (Burdon & Chilvers, 1982). This implies that the epidemics of CLR occurred due to the thousands of coffee trees planted in succession within coffee growing regions in the central Americas. This would exclude the need for an alternate host in order for *H. vastatrix* to proliferate and spread, as the primary host is densely planted and highly accessible to the pathogen. However, this hypothesis does not allow for new variation of the pathogen, but merely maintenance of the clonal propagation of *H. vastatrix*.

The plant pathology community have adopted a somewhat ad hoc approach to identify alternate host plant species, whereby such species are often found serendipitously in disease‐prone environments using a not always structured approach (McDonald, Richardson, Zambino, Klopfenstein, & Kim, 2006; Rodriguez‐Algaba, Walter, Sørensen, Hovmøller, & Justesen, 2014). This has been the case for the alternate host of *Puccinia striiformis* (causal organism of yellow (or stripe) rust of cereal crops), which was recently identified as *Berberis chinensis* after having been unknown for a century (Jin, Szabo, & Carson, 2010). Later, *Berberis vulgaris* was also identified as an alternate host of *P. striiformis* (Rodriguez‐Algaba et al., 2014). Since *B. vulgaris* has been known as the aecial host of *Puccinia graminis* for decades, this may have masked the discovery of *B. vulgaris* as the alternate host of *P. striiformis*. These case studies show the difficulty in determining alternate host species in the context of plant pathology.

Here we present the hypothetical alternate host ranking (HAHR) and automated text mining (ATM) methods to address this gap in knowledge based on a series of assumptions relating to the disease biology of this given pathogen. Our use and integration of comprehensive geographical flora data mapping is novel to traditional plant pathology publications. We believe that this new approach will encourage more multidisciplinary collaborations and hypothesis generation for future studies in this area among plant pathologists and botanists.

## 2 METHOD

**Figure 2:**
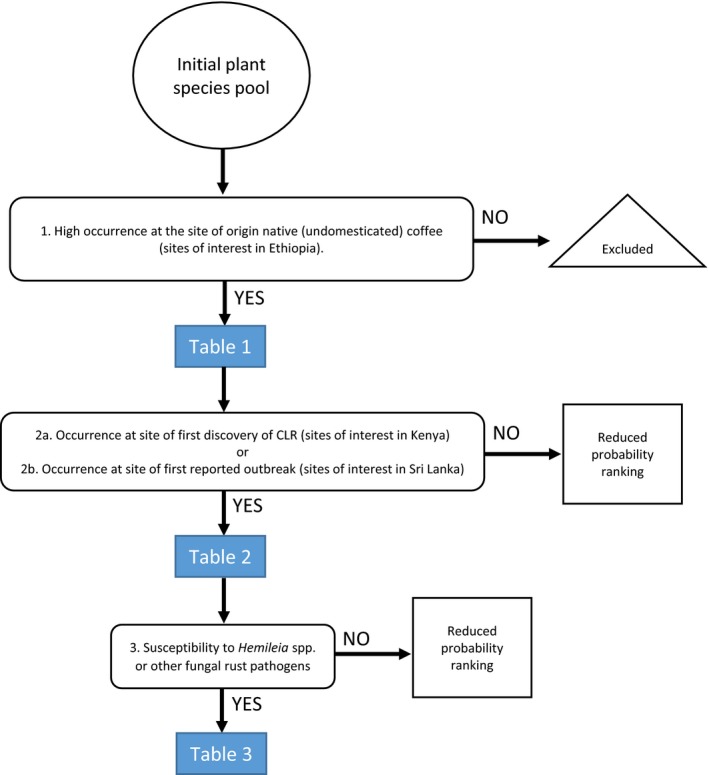
Decision tree used to elude the hypothetical alternate host ranking (HAHR) of *Hemileia vastatrix*

We formulated the so‐called HAHR method in order to create ranked lists of plant species, which could be likely alternate host(s) of *H. vastatrix*. This HAHR takes the form of a decision tree (Figure 2). To begin with, an initial plant species pool was compiled, consisting of 377 different species or genera collated from different sources relating to flora mapping at one of the approximate sites of origin of native (undomesticated) coffee(south‐western highlands of Ethiopia). The sources used included primary literature, online flora databases, and potential vegetation maps. Relevant primary literature was retrieved through a database search using the Web of Science (https://webofknowledge.com/) and Google Scholar (https://scholar.google.dk/) as of 11 June 2019. The following search terms were used both singularly and in combination: “coffee,” “coffea,” “flora mapping,” “vegetation,” “origin,” “Ethiopia,” “Kenya,” and “Sri Lanka.” Plants species and genera listed in over 40 primary sources were then collected and arranged in an MS Excel spreadsheet. The filter function was used to rank plant species or genera according to their co‐occurrence at the site of first discovery of *H. vastatrix* (Lake Kenya region) (Ferreira & Boley, 1991; Waller, 1982) and/or the site of first reported outbreak of CLR (Sri Lanka [Ceylon]) (Berkeley & Broome, 1869). Previous reports of susceptibility to *Hemileia* spp. or other rust pathogens were prioritized in the final ranking. Plant names and authors were verified by The Taxonomic Name Resolution Service (iPlant Collaborative) (available from: http://tnrs.iplantcollaborative.org). Where the name or author match score was less than 100%, The Plant List (http://www.theplantlist.org) was used to crosscheck species. A high‐, medium‐, and low‐ranking list of plant species was then produced based on this HAHR. The rationale for each ranking criterion is described below.

### 2.1 Co‐occurrence with native (undomesticated) *Coffea* spp. at the site of origin

We started by determining the initial plant species pool based on flora mapping studies performed in co‐occurrence of wild *Coffea* spp., specifically in the south‐western highlands of Ethiopia (Gole, 2003; Kelbessa & Soromessa, 2008; Nune, 2008; Schmitt, 2006; Senbeta & Denich, 2006; Tadesse & Nigatu, 1996). Most of the literature found was based on either *Coffea arabica* or nondefined species of wild coffee. As it is by no means certain which species of coffee that *H. vastatrix* co‐evolved with, there is an inherent assumption of origin with *C. arabica* or other unknown wild relatives in our method, based on the literature that was available.

**Figure 3:**
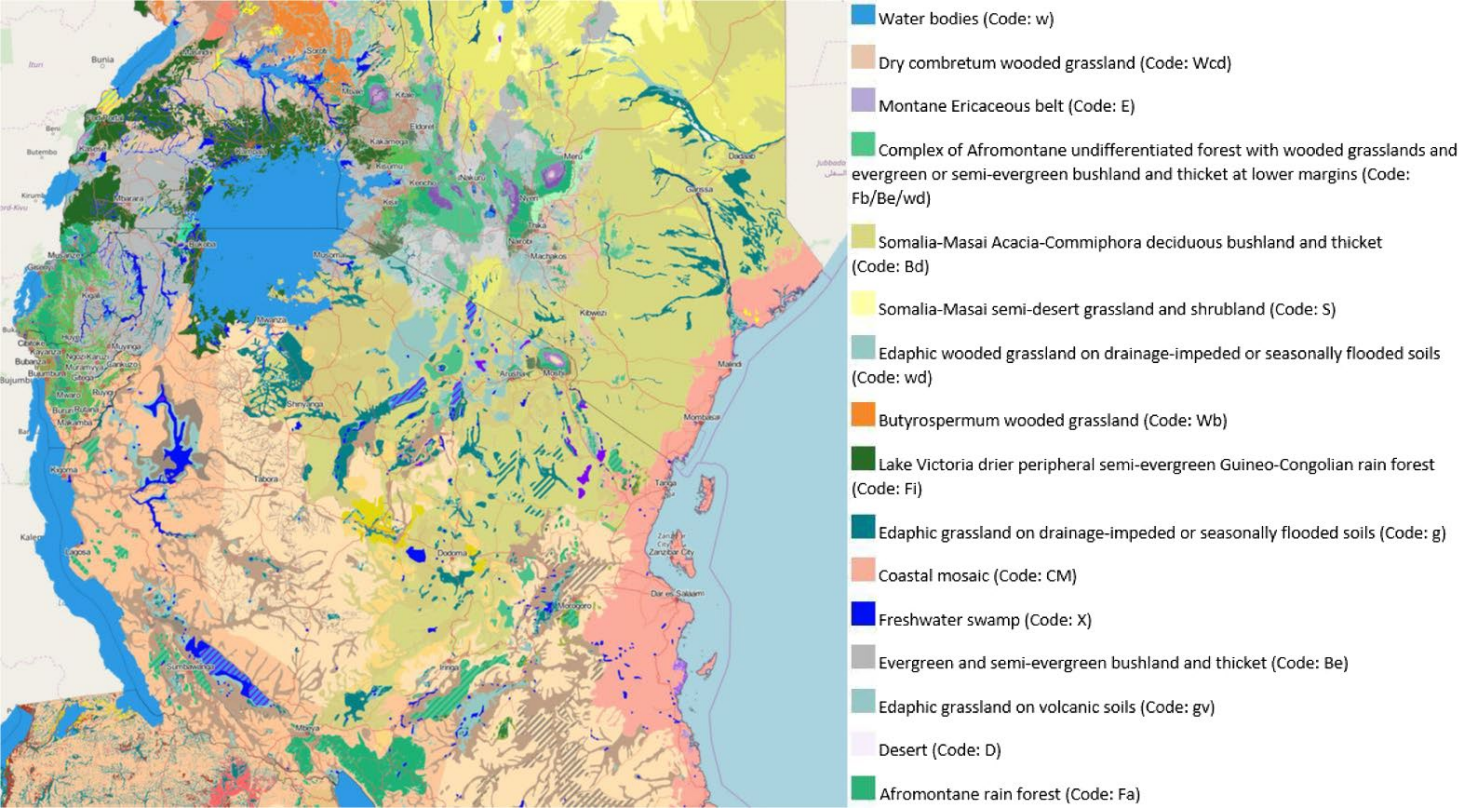
Potential natural vegetation (PNV) map of Ethiopia and Kenya used to define and verify the initial plant species pool (http://maps.vegetationmap4africa.org/). Code regions: Ff, Fa, and Fb/Be/wd were classified as the sites of interest in the hypothetical alternate host ranking method

Potential natural vegetation (PNV) maps of Ethiopia and Kenya were also used to compile the initial plant species pool (Table S1) (van Breugel et al., 2015). PNV maps are defined to illustrate vegetation that would persist under the current climatic conditions without human intervention (van Breugel et al., 2015). The Keffa and Sidamo regions (where the Geba‐Dogi, Berhane‐Kontir, Boginda‐Yeba, and Harenna forest areas lie) have been repeatedly recognized as one of the most probable origins of wild *Coffea* species (Gole, 2003; Meyer, 1965; Schmitt, 2006; Senbeta & Denich, 2006). These regions were collectively assumed as a *site of origin* (or the sites of interest in Ethiopia). According to PNV maps, the sites of interest in Ethiopia were postulated as consisting of either “Complex of Afromontane undifferentiated forest” with “wooded grasslands” or “evergreen or semi‐evergreen bushland and thicket at lower margins” (Figure 3, code: Fb/Be/wd, Kaffa region, Ethiopia) and/or “Afromontane rain forest” (Figure 3, code: Fa, Sidamo region, Ethiopia). The Global Biodiversity Information Facility (https://www.gbif.org/) was used to cross‐reference the primary literature with the PNV map. Plants species, which fulfilled these criteria, are listed in Table 1.

**Table 1:** Low‐ranking list of species considered as potential alternate host(s) of *Hemileia vastatrix*, based on co‐occurrence with the sites of interest in Ethiopia (shown in alphabetical order)

*Acacia abyssinica* Hochst. ex Benth.
*Acacia brevispica* Harms
*Acacia gerrardii* Benth.
*Acacia mellifera* (Vahl) Benth.
*Acacia senegal*(L.) Willd.
*Acacia seyal* Delile
*Acacia sieberiana f. nefasia* (Hochst. ex A. Rich.) Roberty
*Acacia tortilis* (Forssk.) Hayne
*Acacia xanthophloea* Benth.
*Achyranthes aspera* L.
*Afrocanthium keniense* (Bullock) Lantz
*Afrocanthium lactescens* (Hiern) Lantz
*Afrocarpus falcatus* (Thunb.) C.N.Page
*Agarista salicifolia* (Lam.) G.Don
*Alangium chinense* (Lour.) Harms
*Albizia coriaria* Oliv.
*Albizia grandibracteata* Taub.
*Albizia gummifera* (J.F.Gmel.)C.A.Sm.
*Albizia schimperiana* Oliv.
*Albizia zygia* (DC.)J.F.Macbr.
*Alchornea hirtella* Benth.
*Allophylus abyssinicus* (Hochst.) Radlk.
*Allophylus africanus* P.Beauv.
*Allophylus ferrugineus* Taub.
*Anthocleista grandiflora* Gilg
*Apodytes dimidiata* E.Mey. ex Arn.
*Balthasaria schliebenii* (Melch.) Verdc.
*Bersama abyssinica* Fresen.
*Blighia unijugata* Baker
*Bridelia brideliifolia (*Pax) Fedde
*Bridelia micrantha* (Hochst.) Baill.
*Carapa procera* DC.
*Casearia battiscombei* R.E.Fr.
*Cassipourea malosana* (Baker) Alston
*Cassipourea ruwensorensis* (Engl.) Alston
*Catha edulis* (Vahl) Endl.
*Celtis africana* Burm.f.
*Celtis gomphophylla* Baker
*Chrysophyllum gorungosanum* Engl.
*Clausena anisata* (Willd.) Hook.f. ex Benth.
*Cola greenwayi* Brenan
*Cordia africana* Lam.
*Cornus volkensii* Harms
*Crotalaria agatiflora* Schweinf.
*Croton dichogamus* Pax
*Croton macrostachyus* Hochst. ex Delile
*Croton megalocarpus* Hutch.
*Croton sylvaticus* Hochst.
*Cussonia spicata* Thunb.
*Cyathea dregei* Kunze
*Cyathea manniana* Hook.
*Cylicomorpha parviflora* Urb.
*Diospyros abyssinica* (Hiern) F.White
*Dodonaea viscosa* (L.) Jacq.
*Dombeya torrida* (J.F.Gmel.) Bamps
*Dovyalis macrocalyx* (Oliv.) Warb.
*Dracaena fragrans* (L.) Ker Gawl.
*Dracaena steudneri* Engl.
*Drypetes gerrardii* Hutch.
*Ehretia cymosa* Thonn.
*Ekebergia capensis* Sparrm.
*Embelia schimperi* Vatke
*Entandrophragma excelsum* (Dawe & Sprague) Sprague
*Eugenia capensis (*Eckl. & Zeyh.) Harv.
*Euphorbia abyssinica* J.F.Gmel.
*Fagaropsis angolensis* (Engl.) H.M.Gardner
*Ficalhoa laurifolia* Hiern
*Ficus exasperata* Vahl
*Ficus natalensis* Hochst.
*Ficus ovata* Vahl
*Ficus sur* Forssk.
*Ficus thonningii* Blume
*Fleroya rubrostipulata* (K.Schum.) Y.F.Deng
*Galiniera saxifraga* (Hochst.) Bridson
*Garcinia buchananii* Baker
*Grewia arborea* (Forssk.) Lam.
*Grewia damine* Gaertn.
*Grewia ferruginea* Hochst. ex A.Rich.
*Grewia mollis* Juss.
*Grewia similis* K.Schum.
*Grewia tembensis* Fresen.
*Harungana madagascariensis* Lam. ex Poir.
*Ilex mitis* (L.) Radlk.
*Kigelia africana* subsp.* moosa* (Sprague) Bidgood & Verdc.
*Lecaniodiscus fraxinifolius* Baker
*Lepidotrichilia volkensii* (Gürke) J.‐F.Leroy
*Macaranga capensis* (Baill.) Sim
*Maesa lanceolata* Forssk.
*Manilkara butugi* Chiov.
*Margaritaria discoidea* (Baill.) G.L.Webster
*Maytenus acuminata* (L.f.) Loes.
*Maytenus undata (*Thunb.) Blakelock
*Milicia excelsa* (Welw.) C.C.Berg
*Mimusops kummel* Bruce ex A.DC.
*Myrianthus holstii* Engl.
*Neoboutonia macrocalyx* Pax
*Newtonia buchananii* (Baker) G.C.C.Gilbert& Boutique
*Nuxia congesta* R.Br. ex Fresen.
*Ochna holstii* Engl.
*Ocotea kenyensis* (Chiov.) Robyns &R.Wilczek
*Ocotea usambarensis* Engl.
*Olea capensis* L.
*Olea europaea* L.
*Parinari excelsa* Sabine
*Peddiea africana* Harv.
*Pittosporum viridiflorum* Sims
*Plectranthus* spp.
*Podocarpus falcatus* (Thunb.) R. Br. ex Mirb.
*Podocarpus henkelii* Stapf ex Dallim. & B.D.Jacks.
*Podocarpus latifolius* (Thunb.) R.Br. ex Mirb.
*Polyscias fulva* (Hiern) Harms
*Pouteria adolfi‐friedericii* (Engl.) A.Meeuse
*Pouteria altissima* (A.Chev.) Baehni
*Prunus africana* (Hook.f.) Kalkman
*Psychotria mahonii* C.H.Wright
*Psychotria orophila* E.M.A.Petit
*Psydrax parviflora* (Afzel.) Bridson
*Psydrax schimperiana* (A.Rich.) Bridson
*Rapanea melanophloeos* (L.) Mez
*Rhamnus prinoides* L'Heri.
*Ritchiea albersii* Gilg
*Rotheca myricoides* (Hochst.) Steane & Mabb.
*Rothmannia urcelliformis* (Hiern) Bullock ex Robyns
*Rubus apetalus* Poir.
*Schefflera abyssinica* (Hochst. ex A.Rich.) Harms
*Schefflera volkensii* (Harms) Harms
*Scutia myrtina* (Burm.f.) Kurz
*Senna didymobotrya* (Fresen.)H.S.Irwin & Barneby
*Shirakiopsis elliptica* (Hochst.) Esser
*Sinarundinaria alpina* (K. Schum.) C.S. Chao & Renvoize
*Solanum aculeastrum* Dunal
*Strombosia scheffleri* Engl.
*Strychnos mitis* S.Moore
*Symphonia globulifera* L.f.
*Synsepalum brevipes* (Baker) T.D.Penn.
*Syzygium cordatum* Hochst. ex Krauss
*Syzygium guineense* (Willd.) DC.
*Tabernaemontana pachysiphon* Stapf
*Tabernaemontana stapfiana* Britten
*Tarenna graveolens* (S. Moore) Bremek.
*Teclea nobilis* Delile
*Trema orientalis* (L.) Blume
*Trichilia dregeana* Sond.
*Trilepisium madagascariense* DC.
*Turraea holstii* Gürke
*Vachellia drepanolobium* (B.Y. Sjöstedt) P.J.H. Hurter
*Vachellia hockii* (De Wild.) Seigler & Ebinger
*Vachellia kirkii* (Oliv.) Kyal. & Boatwr.
*Vachellia lahai* (Steud. & Hochst. ex. Benth.) Kyal. & Boatwr.
*Vepris dainellii* (Pic. Serm.) Mziray
*Vepris nobilis* (Delile) Mziray
*Vernonia auriculifera* Hiern
*Vernonia myriantha* Hook. f.
*Vitex fischeri* Gürke
*Warburgia salutaris* (G.Bertol.) Chiov.
*Xymalos monospora* (Harv.) Baill.
*Zanthoxylum gilletii* (De Wild.) P.G.Waterman
*Zanthoxylum rubescens* Planch. ex Hook

### 2.2 Co‐occurrence at the site of first discovery of CLR

Subsequently, we analyzed flora mapping at the site of first discovery of CLR. The earliest discovery of CLR was in the Lake Victoria region of Kenya in 1861 by a British explorer on uncultivated, wild coffee (Ferreira & Boley, 1991; Waller, 1982). Given this, we assumed the Lake Victoria region to be the natural site of first discovery of CLR. Based on the PNV maps, the sites of interest in Kenya were hypothesized to be surrounding either the “Lake Victoria transitional rain forest” (Figure 3, code: Ff), “Lake Victoria drier peripheral semi‐evergreen Guineo‐Congolian rain forest” (Figure 3, code: Fi), and/or “Afromontane rain forest” (Figure 3, code: Fa) in close proximity to Kisumu, Kenya. The PNV species web map of eastern Africa (http://vegetationmap4africa.org) was then used to verify the plant species, which occurred concomitantly with the sites of interest in Ethiopia and Kenya. Species which were classified by the PNV species web map as “characteristic” (those documented on at least half of all the national manifestations of the vegetation type) or “present” (those documented to be characteristic in at least one of the national manifestations of the vegetation type) at the sites of interest in Kenya were considered for further analysis. “Marginal” species (those listed on national documentation, but not categorized as characteristic or present) were excluded from the analysis.

### 2.3 Co‐occurrence at the site of first major outbreak of CLR

The first major outbreak of CLR was reported in Sri Lanka (Ceylon) in 1869 (Berkeley & Broome, 1869). After this, CLR spread around the world in three sequential outbreaks (McCook, 2006). From 1870 to 1920, the disease spread through to the Indian Ocean Basin and the Pacific. From 1950 to 1960, West African coffee production was severely affected by CLR. Finally, from the late 1960s onwards, CLR spread across the coffee producing zones throughout the Americas (McCook, 2006).

Transcontinental air dispersion of *H. vastatrix* urediniospores has been reported, but this spore type is more frequently dispersed locally by rain‐splash due to a tendency to adhere strongly to each other, to leaves, and to smooth surfaces (Brown & Hovmøller, 2002; Nutman, Roberts, & Bock, 1960). Thus, the disease severity of CLR has often been associated with heavy rainfall (Waller, 1982). Due to these aspects of spore transmission and the fact that urediniospores have a limited ability to survive on nonliving coffee leaves, we assumed that this first CLR outbreak was the result of the longer‐living teliospores being transported to Sri Lanka on dry plant material. We also assumed that this outbreak was enhanced by the presence of a supportive alternate host in Sri Lanka for the basidiospores to infect, in order to facilitate the generation of new virulent races. Primary flora data (Ashton & Gunatilleke, 1987; Ashton et al., 1997) and The Global Biodiversity Information Facility (https://www.gbif.org/) were used to cross‐reference the plant species or genera present at the sites of interest in Ethiopia, Kenya, and Sri Lanka. Plants, which fulfilled the criteria outlined in the method in Sections 2.2 and 2.3, are listed in Table 2.

**Table 2:** Medium‐ranking list of species considered as potential alternate host(s) of *Hemileia vastatrix*, based on occurrence at the sites of interest in Ethiopia and Kenya, as well as related plant species at the sites of interest in Sri Lanka

Plant species found to occur at the sites of interest in Ethiopia and Kenya^a^	Related plant species found at the sites of interest in Sri Lanka
*Acacia abyssinica* Hochst. ex Benth.	*Acacia decurrens* Willd.
	*Acacia leucophloea* Willd.
	*Acacia mangium* Willd.
	*Acacia melanoxylon* R.Br. in W.T.Aiton
	*Acacia planifrons* Koenig ex Wight & Arn.
*Acacia lahai* Steud. & Hochst. ex Benth.	*Acacia decurrens* Willd.
	*Acacia leucophloea* Willd.
	*Acacia mangium* Willd.
	*Acacia melanoxylon* R.Br. in W.T.Aiton
	*Acacia planifrons* Koenig ex Wight & Arn.
*Albizia coriaria* Oliv.	*Albizia falcataria* (L.) Fosberg
	*Albizia lebbek* sensu auct.
	*Albizia odoratissima* (L.f.) Benth.
	*Albizia saman* (Jacq.) Merr.
*Albizia grandibracteata* Taub.	*Albizia falcataria* (L.) Fosberg
	*Albizia lebbek* sensu auct.
	*Albizia odoratissima* (L.f.) Benth.
	*Albizia saman* (Jacq.) Merr.
*Albizia gummifera* C.A. Sm.	*Albizia falcataria* (L.) Fosberg
	*Albizia lebbek* sensu auct.
	*Albizia odoratissima* (L.f.) Benth.
	*Albizia saman* (Jacq.) Merr.
*Albizia schimperiana* Oliv.	*Albizia falcataria* (L.) Fosberg
	*Albizia lebbek* sensu auct.
	*Albizia odoratissima* (L.f.) Benth.
	*Albizia saman* (Jacq.)Merr.
*Allophylus abyssinicus* (Hochst) Radlk	*Allophylus cobbe* (L.) Raeusch.
*Apodytes dimidiata* E. Mey. ex Arn.	*Apodytes dimidiate* E.Mey. ex Arn.
*Cassipourea malosana* Alston	*Cassipourea* spp.
*Cassipourea ruwensorensis* Alston	*Cassipourea* spp.
*Croton macrostachyus* Hochst. ex Delile	*Croton moonii* Thwaites
*Croton megalocarpus* Hutch.	*Croton moonii* Thwaites
*Croton sylvaticus* Hochst.	*Croton moonii* Thwaites
*Diospyros abyssinica* (Hiern) F.White	*Diospyros ebenum* J.Koenig ex Retz.
	*Diospyros malabarica* (Desr.) Kostel.
	*Diospyros melanoxylon* Roxb.
	*Diospyros oocarpa* Thwaites
	*Diospyros ovalifolia* Wight
*Macaranga capensis* (Baill.) Sim.	*Macaranga indica* Wight
	*Macaranga peltata* (Roxb.) Müll.Arg.
*Ochna holstii* Engl.	*Ochna rufescens* Thwaites
	*Ochna lanceolata* Kuntze
*Psychotria mahonii* Hook. f.	*Psychotria moonii* Hook.f.
*Solanum aculeastrum* Dunal	*Solanum aculeastrum* Dunal(reported in Southern India only)
*Strychnos mitis* S. Moore	*Strychnos nux‐vomica* L.
*Syzygium cordatum* Hochst.	*Syzygium aqueum* (Burm.f.) Alston
	*Syzygium assimile* Thwaites
	*Syzygium cumini* (L.) Skeels
	*Syzygium gardneri* Thwaites
	*Syzygium jambos* (L.) Alston
	*Syzygium lewisii* Alston
	*Syzygium makul* Gaertn.
	*Syzygium neesianum* Arn.
	*Syzygium operculatum* (Roxb.) Nied.
	*Syzygium rubicundum* Wight & Arn.
	*Syzygium umbrosum* Thwaites
	*Syzygium zeylanicum* (L.) DC.
*Syzygium guineense* DC.	*Syzygium aqueum* (Burm.f.) Alston
	*Syzygium assimile* Thwaites
	*Syzygium cumini* (L.) Skeels
	*Syzygium gardneri* Thwaites
	*Syzygium jambos* (L.) Alston
	*Syzygium lewisii* Alston
	*Syzygium makul* Gaertn.
	*Syzygium neesianum* Arn.
	*Syzygium operculatum* (Roxb.) Nied.
	*Syzygium rubicundum* Wight & Arn.
	*Syzygium umbrosum* Thwaites
	*Syzygium zeylanicum* (L.) DC.
*Vitex fischeri* Gürke	*Vitex altissima* L.f.
	*Vitex leucoxylon* L.f.

a Plant species listed were classified as either “characteristic” or “present” at the sites of interest in Ethiopia and Kenya according to the PNV maps. Plants are listed in alphabetical order. Plant names and authors verified by The Taxonomic Name Resolution Service.

### 2.4 Susceptibility to *Hemileia* spp. or other rust fungi

Finally, plant species or genera found concomitantly at the sites of interest in Ethiopia, Kenya, and/or Sri Lanka were also investigated for: (a) any known susceptibility to the *Hemileia* rust species reported (Table S2); and (b) susceptibility to other rust pathogens. This was performed by a review of primary literature obtained through a database search using the Web of Science (https://webofknowledge.com/) and Google Scholar (https://scholar.google.dk/) as of 11 June 2019, plant pathology reference works (Arthur, 1934; Wilson & Henderson, 1966) and the MyCoPortal (http://mycoportal.org). We focused on studies or observations concerning the plant species or genera of interest and any associated rust pathogen. The following search terms were used (in combination with the plant species or genus name): “rust,” “fungi,” “*Hemileia*,” or “fungal pathogen.” All fungal names and authors were verified by Species Fungorum (http://www.speciesfungorum.org/). Table 3 is made based on the presence of the plant species or genera at the site of origin (Ethiopia) and/or the site of first discovery (Kenya) or first outbreak (Sri Lanka) as well as association with (a) *Hemileia* spp. and (b) other rust fungi. The different rust spore stages were classified according to Hiratsuka and Sato (1982):

Spore stage 0: Spermogonia (consists of receptive hyphae and spermatia)
Spore stage I: Aeciospores in aecia
Spore stage II: Urediniospores in uredia
Spore stage III: Teliospores in telia
Spore stage IV: Basidiospores on basidia

**Table 3:** High‐ranking list of species considered as potential alternate host(s) of *Hemileia vastatrix*, based on the hypothetical alternate host ranking (HAHR)

Plant species^a^	The sites of interest	Known interactions with *Hemileia* spp. or other rust pathogens
Category 1 ranking		
*Psychotria mahonii* C.H.Wright	E, K & S^b^	Genus is susceptible to *Hemileia holstii* P. Syd. & Syd. (MyCoPortal, 2018)
*Rhamnus prinoides* L'Heri	E, K & S^b^	Genus is susceptible to rust fungus: *Puccinia coronata* Peturson reported on *Rhamnus* spp. in the spermogonial (0) and aecial rust stages (I) (Arthur, 1934 pp. 152; Nazareno et al., 2017)
*Rubus apetalus* Poir.	E, K & S^b^	Species and genus are susceptible to rust fungus: *Kuehneola uredinis* (Link) Arthur in either the uredinial (II) and telial (III) rust stages (MyCoPortal, 2018; Van Reenen, 1995)
Category 2 ranking		
*Antidesma venosum E.Mey. ex Tul*.	E, K	Genus is susceptible to *Hemileia antidesmae* P. Syd. & Syd. (MyCoPortal, 2018)
*Canthium keniense* Bullock	E, S^b^	Genus is susceptible to *Hemileia canthi* (Berk. & Broome) in the spermogonial (0) and aecial rust stages (I) (MyCoPortal, 2018)
*Canthium lactescens* Hiern	E, S^b^	Genus is susceptible to *Hemileia canthi* (Berk. & Broome) in the spermogonial (0) and aecial rust stages (I) (MyCoPortal, 2018)
*Canthium oligocarpum* Hiern	E, S^b^	Genus is susceptible to *Hemileia canthi* (Berk. & Broome) in the spermogonial (0) and aecial rust stages (I) (MyCoPortal, 2018)
*Clerodendrum myricoides* (Hochst.) R.Br. ex Vatke	E, S^b^	Genus is susceptible to *Hemileia scholzii* Syd. & P. Syd. (MyCoPortal, 2018)
*Harungana madagascariensis* Poir.	E, K	Species is susceptible to *Hemileia harunganae* Cummins (MyCoPortal, 2018)
*Vangueria apiculata* K. Schum.	E, K	Genus is susceptible to *Hemileia thomasii* Thirum. & Naras. and Hemileia woodii Kalchbr. & Cooke
Category 3 ranking		
*Alchemilla adolfi‐friederici* Engl.	E, K	Genus is susceptible to *Trachyspora alchemillae* (Pers.) Fuckel in the uredinial rust stage (II) (Helfer, 2005; MyCoPortal, 2018) and reported susceptibility to decay by basidiomycete fungi (Desalegn, 2013)
*Cornus volkensii* Harms	E, K	Genus is susceptible to rust fungus: *Puccinia porphyrogenita* M.A. Curtis reported on *Cornus canadensis* in either the uredinial (II) or telial (III) rust stages (Arthur, 1934 pp. 251; MyCoPortal, 2018)
*Croton dichogamus* Pax	E, S^b^	Genus is susceptible to rust fungus: *Bubakia crotonis* Arthur reported on *Croton argyranthemus*, *Croton californicus*, *Croton capitatus*, *Croton engelmannii*,* Croton monanthogynus*,* Croton punctatus*, and *Croton texenis* in either the uredinial (II) or telial (III) rust stages (Arthur, 1934 pp. 60; MyCoPortal, 2018)
*Croton macrostachyus* Hochst. ex Delile	E, S^b^	Genus is susceptible to rust fungus: *Bubakia crotonis* Arthur reported on *Croton argyranthemus*, *Croton californicus*, *Croton capitatus*, *Croton engelmannii*,* Croton monanthogynus*,* Croton punctatus*, and *Croton texenis* in either the uredinial (II) or telial (III) rust stages (Arthur, 1934 pp. 60; MyCoPortal, 2018)
*Croton sylvaticus* Hochst.	E, S^b^	Genus is susceptible to rust fungus: *Bubakia crotonis* Arthur reported on *Croton argyranthemus*,* Croton californicus*, *Croton capitatus*, *Croton engelmannii*,* Croton monanthogynus*,* Croton punctatus*, and *Croton texenis* in either the uredinial (II) or telial (III) rust stages (Arthur, 1934 pp. 60; MyCoPortal, 2018)
*Euphorbia abyssinica* J.F.Gmel.	E, K	Genus is susceptible to rust fungus: *Melampsora euphorbiae* (Ficinus & C. Schub.) Castagne is found on a wide range of Euphorbia spp. in either I, II, III, or 0 rust stages (Wilson & Henderson, 1966 pp. 68 and Arthur, 1934 pp.309; MyCoPortal, 2018)
*Ficus ovata* Vahl	E, K	Genus is susceptible to rust fungus: *Physopella fici* (Castagne) Arthur reported on *Ficus carica* in either the uredinial (II) or telial (III) rust stages (Arthur, 1934 pp. 61; MyCoPortal, 2018)
*Ficus sur* Forssk.	E, K	Genus is susceptible to rust fungus: *Physopella fici* (Castagne) Arthur reported on *Ficus carica* in either the uredinial (II) or telial (III) rust stages (Arthur, 1934 pp. 61; MyCoPortal, 2018)
*Ficus thonningii* Blume	E, K	Genus is susceptible to rust fungus: *Physopella fici* (Castagne) Arthur reported on *Ficus carica* in either the uredinial (II) or telial (III) rust stages (Arthur, 1934 pp. 61; MyCoPortal, 2018)

a The plant species or the genera were found to occur at the sites of interest in Ethiopia, Kenya, and/or Sri Lanka and have a known susceptibility to Hemileia spp. or other rust pathogens. Plants are listed in a prioritized category ranking order. Category 1 ranking implies that the plant species or genera have been observed at all the sites of interest and have a reported susceptibility to Hemileia spp. or other rust pathogens. Category 2 ranking implies that the plant species or genera have been observed at two sites of interest and have a known susceptibility to a Hemileia species. Category 3 ranking implies that the plant species or genera have been observed at two sites of interest and have a known susceptibility to other rust pathogens. Plant names and authors verified by The Taxonomic Name Resolution Service. Fungi names and authors verified by Species Fungorum. The sites of interest are stated as E (Ethiopia), K (Kenya), and S (Sri Lanka).

b Only present at genus level.

### 2.5 Automated text mining (ATM) approach

Automated text mining of the biomedical literature has been widely used to recognize entities such as species, proteins, or diseases in the scholarly literature, for example, (Pafilis et al., 2013; Piñero et al., 2015). To our knowledge, this is the first attempt to apply this methodology in the context of plant flora data. Dictionary‐based text mining uses a fixed set of identifiers and synonyms that are matched to the contents of scientific articles to identify articles mentioning an entity of interest.

We used text mining to identify species that are comentioned with *C. arabica* in an automated manner. We used the ORGANISMS web resource (Pafilis et al., 2013) to programmatically identify taxa comentioned with *C. arabica* (NCBI Taxonomy ID: 13443) in PubMed abstracts. ORGANISMS recognizes terms from the NCBI Taxonomy via ATM and is updated weekly. We used a version of ORGANISMS downloaded on 9 April 2019. Using a custom Python script, we first identified all PubMed abstracts mentioning *C. arabica* from the ORGANISMS download files. This set of abstracts was then intersected with the set of abstracts mentioning each taxon to determine the number of abstracts comentioning *C. arabica* and the respective taxon. Since the ORGANISMS web resources uses fully automated pattern matching to identify taxa from the NCBI Taxonomy in PubMed abstracts, the mentions contained in ORGANISMS may contain both false‐positive matches (the taxon is falsely recognized in the abstract) and false‐negative matches (the taxon is falsely overlooked in the abstract). Any plant species that are found in the NCBI taxonomy lineage for *C. arabica* are automatically listed as a comentioned item in the ATM results (https://www.ncbi.nlm.nih.gov/Taxonomy/Browser/wwwtax.cgi?xml:id=13443).

### 2.6 Comparison of the HAHR and the ATM methods

A manual cross‐referencing approach was applied to the list of species generated by the ATM method by using the “find” function in MS Excel for all of the plant species listed in the HAHR method (Tables 1, 2, 3). The ranking established in the HAHR was also applied to the ATM (Table 4). The abstracts listed by the ATM method were reviewed to assess whether both *C. arabica* and the potential plant host species were evident in the publication and how many times they were comentioned (Table 4). The percentage difference in overlap of potential host species was calculated as a way to compare the HAHR and ATM methods output.

**Table 4:** Cross‐reference of high‐, medium‐, and low‐ranking species with the automated text mining method (ATM)

Plant genus	Number of abstracts comentioned with *C. arabica*
High‐ranking list	
*Rubus* spp.	5
*Croton* spp.	3
*Euphorbia* spp.	8
Medium‐ranking list	
*Acacia* spp.	8
*Solanum* spp.	49
*Syzygium* spp.	6
Low‐ranking list	
*Vachellia* spp.	2
*Synsepalum* spp.	2
*Prunus* spp.	11
*Pittosporum* spp.	2
*Olea* spp.	5
*Ocotea* spp.	1
*Maytenus* spp.	2
*Garcinia* spp.	3
*Cyathea* spp.	1
*Cornus* spp.	3
*Cordia* spp.	1
*Catha* spp.	2
*Alchornea* spp.	2

## 3 RESULTS AND DISCUSSION

The HAHR method indicated 158 plant species as potential alternate hosts of *Hemileia vastarix*, while the ATM method listed over 2,179 species (although some duplication was found). There were 19 plant species, which overlapped both methods (Table 4). Indicating that 12% of the HAHR findings were corroborated by the ATM method. The low overlap percentage reflects the variation in the two methods.

The HAHR method produced a low‐ranking short list (Table 1), a medium‐ranking list (Table 2), and a high‐ranking list (Table 3) of plant species. Table 1 comprises 158 plant species, which were found at the sites of interest in Ethiopia and Kenya in flora mapping studies concomitantly with *Coffea* species. The absence of common species between the sites of interest in Ethiopia, Kenya, and Sri Lanka (Table 2) could be seen as an indication that there would be more than one possible alternate host species of CLR. High rates of endemism caused by the long period of isolation of the island (Gunatilleke & Gunatilleke, 1990) mean that over a quarter of the native species present in Sri Lanka are considered unique to the country (Ashton et al., 1997). However, the Deccan‐Gondwana ancestry of the country means there may have been an early contribution to the natural plant communities of Sri Lanka's west coast from the African continent (Ashton et al., 1997). This may explain why there are similarities at the genus level at the sites of interest in Ethiopia, Kenya, and Sri Lanka (Table 2).

Table 3 shows 22 high‐ranking, potential alternate hosts of *H. vastatrix*, belonging to 10 different families (Cornaceae, Euphorbiaceae, Hypericaceae, Lamiaceae, Moraceae, Phyllanthaceae, Podocarpaceae, Rhamnaceae, Rosaceae, and Rubiaceae). This list includes conifers and angiosperm dicots such as succulents, herbs, shrubs, and trees. Table 3 has been divided into three additional categories of ranking. Category 1 ranking implies that the plant species or genera have been observed at all the sites of interest and has a reported susceptibility to *Hemileia* spp. or other rust pathogens. Category 2 ranking implies that the plant species or genera have been observed at two sites of interest and have a known susceptibility to a *Hemileia* species. Category 3 ranking implies that the plant species or genera have been observed at two sites of interest and have a known susceptibility to other rust pathogens. The highest‐ranking plant species found were *Psychotria mahonii*, *Rubus apetalus*, and *Rhamnus prinoides*. All three species were present at the sites of interest in Ethiopia and Kenya as well as having a presence at the genus level in Sri Lanka. Furthermore, these plant species are known hosts of *Hemileia holstii* (MyCoPortal, 2018), *Kuehneola uredinis* (Van Reenen, 1995), and *Puccinia coronata* (Nazareno et al., 2017), respectively.

Overall, minimal botanical data were found for *P. mahonii*. This plant species is stated as being either a tree (up to 15 m) or shrub (up to 5 m) and native to Burundi, Cameroon, Gabon, Kenya, Malawi, Rwanda, Sudan, Tanzania, Uganda, Zambia, Zaïre, and Zimbabwe (Flora of Tropical East Africa, 2018). Given that *Psychotria* spp. are native and widespread across the tropics and subtropical regions (including Sri Lanka), the likelihood of interaction with *H. vastatrix* is high and therefore makes this species a high‐ranking candidate as an alternate host of the fungus.

*Rubus apetalus* has been documented as a host of the rust fungus *Kuehneola uredines* (casual pathogen of blackberry cane and leaf rust) in the uredinial stage (Van Reenen, 1995). Interestingly, the telial, spermogonial, and aecial states of *K. uredinis* have also been observed on *Rubus penetrans* (Gardner & Hodges, 1983), thus making this plant species a possible aecial host for other rust fungi. Similarly, *R. prinoides* is the alternate host of *Puccinia coronate* (causal pathogen of crown rust in cultivated and wild oat) (Nazareno et al., 2017). *Rhamnus* and *Rubus* spp. are known for occurring pervasively across a wide range of habitats including mountain forests, especially in clearings and along edges; along watercourses; in riverine forests; in margins of evergreen forests; in secondary mountain evergreen forests or bushes; on mountain slopes; and in grasslands (http://ecocrop.fao.org; Ruffo, Birnie, & Tengnäs, 2002). Both the genera *Rhamnus* and *Rubus* are widespread throughout Asia, Africa, and the Americas, which are today coffee leaf rust hotspots.

The ATM method recovered 700 articles, which mentioned *C. arabica* and 2,179 taxa were comentioned at least once with *C. arabica*. These taxa included plants, animals, fungi, and bacteria. The 2,177 taxa were sorted by the number of PubMed abstracts that comention them with *C. arabica*. The relevant plant genera derived from the ATM and manual analysis are shown in Table 4. Several genera have been comentioned with *C. arabica* in the PubMed databases. The cross‐referenced results by the two methods suggest that plant genera of interest are Rubus, Croton, and Euphorbia. Rubus is of high interest as it is the only commonly found genus in both the category one ranking of the HAHR and with the ATM method. The genus Solanum was very often comentioned with *C. arabica* (49 abstracts) according to the ATM method. However, it was only listed on the medium‐ranking list when using the HAHR method. This shows the variation between the two methods as the HAHR is based on published flora data showing co‐occurrence at the sites of interest, whereas the ATM shows search results of *C. arabica* plus any other comentioned species independent of the geography or nature of the published study.

Both the methods used in this hypothesis paper are limited by the published literature and databases, which were used as the “data pool” for each of the analyses. The HAHR method relies on published flora data produced in English and is region‐specific based on the decision tree (Figure 2) making it a targeted method in the context of this study. On the other hand, the ATM method is restricted to the PubMed databases and includes all species (animals, bacteria, fungi, and plants) comentioned with *C. arabica*, making it more comprehensive than the HAHR method. However, this also leads to superfluous data retrieval, which needed to be manually filtered. Furthermore, the ATM method may yield false‐positive hits due to the species names listed in the NCBI taxonomy, that are falsely recognized in the analyzed articles. Again, manual filtering avoided these false‐positive results to be included in the results. Given the incorporation of plant species geography into the HAHR method, it is recommended that the findings from this method be prioritized over the ATM method.

A corroboration of the hypothesis raised in this study would be morphological and molecular examinations of historical plant leaf samples from herbaria collections. Plant species from the ranked listings (Tables 1, 2, 3), which were collected during epidemic periods, may exhibit symptoms of CLR, which can help to verify the alternate host. This approach has led to the recent revision of the history and geographical range of *Colletotrichum acutatum* species (Sundelin et al., 2015), as well as the sequencing of a unique genotype of *Phytophthora infestans* (HERB‐1), which is now accepted as the causal virulent race which lead to the 19th century potato late blight epidemic (Yoshida et al., 2013).

Based on the HAHR and ATM methods presented here, it is the hope that the alternate host(s) of *H. vastatrix* will be conclusively identified. Priority for future exploratory studies in this context should be offered to plant species *P. mahonii*, *R. apetalus*, and *R. prinoides*, as indicated by the HAHR method. Further exploration of the genera *Croton*,* Euphorbia*, and *Rubus*, as indicated by the cross‐referencing with the ATM method, is also of interest. The ranked plant species lists presented here may be used as a guide to search for signs of spermogonia and aecia of *H. vastatrix* under natural infection conditions or on historical specimens, from the areas we have presented as the sites of origin, discovery or first reported outbreak of CLR. By narrowing down the possible alternate host of *H. vastatrix*, it is our hope to help solve the mystery that has been perplexing the plant pathology community for more than 150 years.

## CONFLICT OF INTEREST

The authors declare that they have no competing interests.

## AUTHOR CONTRIBUTIONS

AK was responsible for the HAHR analysis and manual ATM analysis and for developing the draft. AR and JPBL contributed to the initial plant species pool development and flora literature review. HJLJ and BJ contributed to the literature review concerning the pathogen biology and history. AJ was responsible for the adaptation to the ATM method. All authors commented on the manuscript and approved the final version. None of the authors has any competing interests in the manuscript.

## ETHICS APPROVAL AND CONSENT TO PARTICIPATE

Not applicable

## CONSENT FOR PUBLICATION

Not applicable

## Data Availability

Databases generated or analyzed during this study are included in this published article [and its supplementary information files].
