# Supplementary material for: On the hunt for the alternate host of Hemileia vastatrix
Source: Ecol Evol. 2019 Nov 23;9(23):13619–31. doi: 10.1002/ece3.5755 (PMC6912922; doi:10.1002/ece3.5755)
Supplement: Supplementary file 2 [file ECE3-9-13619-s002.docx]

**Supplementary Table 2.** List of fungal *Hemileia* species and reported host plant(s) in alphabetical order.

| *Hemileia* spp. | Authorship | Observed rust spore stage | Reported host plant(s) |
| --- | --- | --- | --- |
| *Hemileia alafiae* | (Cummins & Gopalkr.) Judith & Rossman | - | *Alafia microstylis* |
| *Hemileia americana* | Massee | - | Unknown |
| *Hemileia antidesmae* | P. Syd. & Syd. | - | *Antidesma bunius* |
| *Hemileia aureospora* | J.M. Yen | - | *Sabicea discolor* |
| *Hemileia buntingii* | Wakef. & Hansf. | - | *Strophanthus hispidus* and *Strophanthus sarmentosus* |
| *Hemileia canthii* | Berk. & Broome | 0 & I | *Canthium ciliatum, Canthium gynochthodes, Canthium parviflorum, Canthium pedunculare, Plectronia gynochthodes, Plectronia horrida, Plectronia pedunculari, Plectronia setiflora* and *Psydrax subcordata* |
| *Hemileia chlorocodonis* | Syd. & P. Syd. | II, III | *Apocynaceae* spp.*, Chlorocodon wightii, Tacazzea apiculata* |
| *Hemileia coffeicola* | Maubl. & Roger | - | *Coffea arabic, Coffea laurin and Craterispermum laurinum* |
| *Hemileia deightonii* | Syd. | - | *Rauwolfia vomitoria* |
| *Hemileia dioscoreae-aculeatae* | (Racib.) P. Syd. & Syd. | - | *Dioscorea aculeata and Dioscorea bulbifera* |
| *Hemileia evansii* | Syd. & P. Syd. | - | *Tricalysia sonderiana* |
| *Hemileia fadogiae* | (Henn.) Syd. & P. Syd. | II | *Fadogia cienkowski, Fadogia stenophylla odorata and Rubiaceae* |
| *Hemileia gardeniae-floridae* | Sawada | - | *Gardenia lutea* |
| *Hemileia gardeniae-thunbergiae* | (Henn.) Maubl. & Roger | II | *Gardenia latifolia, Gardenia lutea, Gardenia thunbergi, Gardenia volkensii* |
| *Hemileia hansfordii* | Syd. | - | *Jasminum abyssinicum, Jasminum dichotomum and Jasminum pubescens* |
| *Hemileia harunganae* | Cummins | - | *Harungana madagascariensis* |
| *Hemileia helvola* | Syd. & P. Syd. | - | Unknown |
| *Hemileia holarrhenae* | Syd. & P. Syd. | II | *Holarrhena antidysenterica* and *Wrightia antidysenterica* |
| *Hemileia holstii* | P. Syd. & Syd. | - | *Psychotria nigropunctat, Psychotria orophila* and *Rubiaceae* |
| *Hemileia jahnii* | Syd. | - | *Mesechites trifidus* |
| *Hemileia jasmini* | C.S. Krishnam. & Rangaswami | - | *Jasminum ritchiei* |
| *Hemileia kumasensis* | (Cummins) Judith & Rossman | - | *Sabicea sp.* |
| *Hemileia mandevillae* | Cummins | - | *Mandevilla sp.* |
| *Hemileia mildbraedii* | (Syd. & P. Syd.) P. Syd. & Syd. | - | *Pavetta ternifolia* |
| *Hemileia myathaungii* | Thaung | - | *Catunaregam spinosa aff.* |
| *Hemileia mysorensis* | Thirum. & Gopalakrishn. | - | *Gymnema sp.* |
| *Hemileia oncidii* | Griffon & Maubl. | - | *Oncidium lanceanum* and *Oncidium rogersii* |
| *Hemileia oxyanthi* | Cummins | - | *Oxyanthus speciosus* |
| *Hemileia pavetticola* | Maubl. & Roger | - | *Pavetta sp.* |
| *Hemileia phaji* | (Racib.) P. Syd. & Syd. | - | *Dendrobium chrysotoxum, Phaius grandifolius* and *Plocoglottis sakiensis* |
| *Hemileia pieningii* | Deighton | - | *Mussaenda erythrophylla* |
| *Hemileia ruspoliae* | Cummins | - | *Ruspolia hypocrateriformis* |
| *Hemileia rutideae* | Cummins | - | *Keetia gueinzii* and *Rutidea rufipilis* |
| *Hemileia scholzii* | Syd. & P. Syd. | - | *Clerodendrum buchholzii, Clerodendrum capitatu, Clerodendrum glabrum, Clerodendrum johnstonii, Clerodendrum scandens* and *Clerodendron spelendens* |
| *Hemileia scitula* | Syd. | - | *Periploca nigrescens* |
| *Hemileia smalliana* | Gjaerum | - | *Strophanthus courmontii* and *Strophanthus sarmentosus* |
| *Hemileia smallii* | Wakef. & Hansf. | - | *Strophanthus sarmentosus* |
| *Hemileia strophanthi* | Racib. | - | *Strophanthus dichotomus* and *Strophanthus sarmentosus* |
| *Hemileia sydowiorum* | Ritschel | - | *Pavetta ternifolia* |
| *Hemileia thomasii* | Thirum. & Naras. | - | *Gardenia jovis* and *Vangueria spinosa* |
| *Hemileia vastatrix* | Berk. & Broome | I, II & III | *Coffea arabica L., Coffea bengalensis, Coffea canephora, Coffea liberica, Coffea stenophylla* and *Coffea benghalensis* |
| *Hemileia voacangae* |  | - | *Voacanga africana* |
| *Hemileia woodii* | Kalchbr. & Cooke | - | *Gardenia gummifera, Pachystigma latifolium, Vangueria edulis, Vangueria infausta, Vangueriopsis lanciflora* and *Vangueria madagascariensis* |
| *Hemileia wrightiae* | (Racib.) Racib. | II & III | *Wrightia arborea, Wrightia laniti, Wrightia mollissima, Wrightia pubescens laniti* and *Wrightia tinctoria* |
